# Deep-learning-based 3D super-resolution MRI radiomics model: superior predictive performance in preoperative T-staging of rectal cancer

**DOI:** 10.1007/s00330-022-08952-8

**Published:** 2022-06-21

**Authors:** Min Hou, Long Zhou, Jihong Sun

**Affiliations:** Department of Radiology, Sir Run Run Shaw Hospital, Zhejiang University School of Medicine, No. 3 East Qingchun Road, Hangzhou, 310016 Zhejiang Province China

**Keywords:** Rectal cancer, Radiomics, Super-resolution, Magnetic resonance imaging, Preoperative T-staging

## Abstract

**Objectives:**

To investigate the feasibility and efficacy of a deep-learning (DL)-based three-dimensional (3D) super-resolution (SR) MRI radiomics model for preoperative T-staging prediction in rectal cancer (RC).

**Methods:**

Seven hundred six eligible RC patients (T1/2 = 287, T3/4 = 419) were retrospectively enrolled in this study and chronologically allocated into a training cohort (*n* = 565) and a validation cohort (*n* = 141). We conducted a deep-transfer-learning network on high-resolution (HR) T2-weighted imaging (T2WI) to enhance the *z*-resolution of the images and acquired the preoperative SRT2WI. The radiomics models named model_HRT2_ and model_SRT2_ were respectively constructed with high-dimensional quantitative features extracted from manually segmented volume of interests of HRT2WI and SRT2WI through the Least Absolute Shrinkage and Selection Operator method. The performances of the models were evaluated by ROC, calibration, and decision curves.

**Results:**

Model_SRT2_ outperformed model_HRT2_ (AUC 0.869, sensitivity 71.1%, specificity 93.1%, and accuracy 83.3% vs. AUC 0.810, sensitivity 89.5%, specificity 70.1%, and accuracy 77.3%) in distinguishing T1/2 and T3/4 RC with significant difference (*p* < 0.05). Both radiomics models achieved higher AUCs than the expert radiologists (0.685, 95% confidence interval 0.595–0.775, *p* < 0.05). The calibration curves confirmed high goodness of fit, and the decision curve analysis revealed the clinical value.

**Conclusions:**

Model_SRT2_ yielded superior predictive performance in preoperative RC T-staging by comparison with model_HRT2_ and expert radiologists’ visual assessments.

**Key Points:**

*• For the first time, DL-based 3D SR images were applied in radiomics analysis for clinical utility.*

*• Compared with the visual assessment of expert radiologists and the conventional radiomics model based on HRT2WI, the SR radiomics model showed a more favorable capability in helping clinicians assess the invasion depth of RC preoperatively.*

*• This is the largest radiomics study for T-staging prediction in RC.*

**Supplementary Information:**

The online version contains supplementary material available at 10.1007/s00330-022-08952-8.

## Introduction

Currently, colorectal cancer ranks 3^rd^ and 2^nd^, respectively, in the global oncological incidence and death spectrum [1, 2]. Rectal cancer (RC) accounts for 1/3 of colorectal cancer [3] and has the highest incidence in East Asia [4]. Although radical surgery remains the only cure, the administration of neoadjuvant chemoradiotherapy (nCRT) that was based on pretreatment staging has increased the R0 resection rate and improved local control [5–7]. nCRT is recommended as standard therapy for locally advanced RC (LARC) patients (T3/4 and/or N+), while it is unnecessary for early-stage patients (T1/2 and N−) [8, 9]. To avoid over- or under-treatment, accurate assessment of preoperative T-staging to distinguish T1/2 from T3/4 tumors is necessary. High-resolution (HR) T2-weighted imaging (T2WI) with fine soft tissue resolution serves as the first-line imaging modality for local staging of RC [10]; however, the overall accuracy ranges from 60 to 75% [11–14], and previous studies have reported a mean overstaging rate of 30–57% for T2 tumors [15, 16]. In this case, it is of great clinical value to improve the assessment of primary tumor T-staging for precise treatment and optimal therapeutic strategy.

As a flourishing novel approach, radiomics introduces a noninvasive way to quantitatively evaluate tumor heterogeneity through mining high-dimensional data from medical images [17]. Previous studies have suggested its potential to improve patient management and clinical decision-making by uncovering disease characteristics that may be invisible to human eyes [18, 19]. In RC, radiomics has attained impressive performance in different oncological scenarios, including evaluating tumor biological behaviors [20], assessing treatment response [21, 22], and predicting prognosis [23]. Despite these advances, radiomics features tend to be affected by anisotropic resolution and low voxel statistics in current medical imaging [24]. To enhance the robustness and stability of radiomics models, it is considerable to tackle these limitations by applying higher-resolution images in model construction.

The super-resolution (SR) technique, aiming at recovering higher spatial resolution of digital images from lower-resolution observations, has been come up with since the 1980s [25]. In recent years, with the development of deep learning (DL), SR has achieved superior performance in medical imaging [26]. As for its application in MRI examinations, Masutani et al [27] proposed a novel convolutional neural network (CNN) model which optimized high-frequency spatial detail in short-axis cardiac MRI imaging; in the work named SCSRN, Niaz et al [28] reported that DL-SR algorithms improved the image quality for brain morphology diagnosis. Apart from the good stability and reliability the recovered SR images showed in the multi-space-ladder, DL-SR has attracted attention in medical imaging because the radiomics features extracted from SR images were quantitatively proven to be remarkably reproducible and robust [24]. However, none of the current radiomics researches have utilized the DL-SR technique in the discovery of a specific radiomics biomarker for clinical use. Here, we aimed to develop and validate a SR radiomics model for preoperatively predicting the T-staging of RC patients. To the best of our knowledge, this study is the first to demonstrate DL-based three-dimensional (3D) SR radiomics in clinical settings.

## Material and methods

### Patients

The retrospective study was approved by the institutional review board with a waiver of written informed consent. Consecutive patients from two centers in our institution with pathologically confirmed rectal adenocarcinoma between January 2014 and October 2020 were retrospectively enrolled in this study.

Inclusion criteria were as follows: (1) patients with definite pathological T-staging information from radical surgery; (2) pelvic MRI within 2 weeks before surgery; (3) no preoperative treatment; (4) single lesion and absence of distant metastases confirmed by imaging techniques or clinical examinations; and (5) available medical records.

Exclusion criteria were (1) lesions invisible on HRT2WI or too tiny for segmentation; (2) a history of pelvic surgery or concurrent with other malignancy; and (3) insufficient image quality for T-staging diagnosis or feature extraction.

**Fig. 1:**
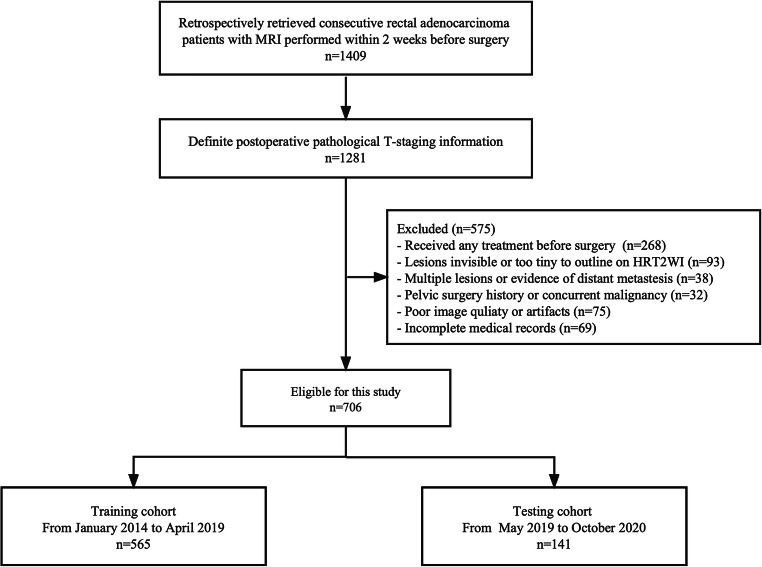
Flowchart of patient recruitment in this study

Finally, 706 eligible patients were chronologically divided into a training cohort (*n* = 565, from January 2014 to April 2019) and a validation cohort (*n* = 141, from May 2019 to October 2020) at a proportion of 8:2. The study is schematically presented in Fig. 1. Demographical information, laboratory tests, and clinical characteristics were derived from electronic medical records.

### Pathological evaluation

The specimen sections were subjected to hematoxylin-eosin staining after fixation in formalin solution for 48 h. All histopathological results were evaluated and determined by two specialist gastrointestinal pathologists who were blinded to the patients’ history. The histopathology reports compromised local staging, tumor deposits, perineural invasion (PNI), lymphovascular invasion (LVI), and extramural venous invasion (EMVI) status. Tumor size, described as the maximal diameter on the maximal cross section of the specimen, was measured by the two observers and a mean value was recorded. The (yp)T stage was classified into four categories according to the 8^th^ edition of the American Joint Commission on Cancer (AJCC) TNM classification [29] as follows: T1, T2, T3, and T4 (Supplementary materials 1).

### Image acquisition and evaluation

Original MRI scans were performed in the supine position at two centers in our institution on one of the two 3.0-T MR scanners (GE Signa HDx, GE Healthcare; Siemens MR Skyra, Siemens Healthineers) with a 16-channel phase-array body coil. Patients fasted for 4 h and emptied their bowels before examination. A routine mp-MRI protocol including a combination of oblique axial HRT2WI, sagittal T2WI, axial DWI (*b* value = 0, 800 s/mm^2^), axial T1-weighted imaging (T1WI), and gadolinium contrast-enhanced T1WI of the pelvis was performed for all patients. The oblique axial scan was performed perpendicular to the long axis of the tumor. Detailed HRT2WI acquisition parameters are shown in Table 1.

**Table 1:** MRI acquisition parameters for HRT2WI protocols

Acquisition parameters	GE Signa HDx	Siemens MR Skyra
Magnetic field strength	3.0 T	3.0 T
Sequence name	FSE	TSE
Repetition time (ms)	6538	4000
Echo time (ms)	130	108
Flip angle (°)	110	160
Field of view (mm × mm)	160 × 160	160 × 160
Scan matrix	512 × 512	512 × 512
Voxel size (mm)	0.312*0.312*3.300	0.312*0.312*3.300
Slice thickness (mm)/gap(mm)	3.0/0.3	3.0/0.3
Slices	20	20
Echo train length	20	16
Bandwidth (Hz)/FA (°)	62.5/110	108/160

*HRT2WI* high-resolution T2-weighted imaging

**Fig. 2:**
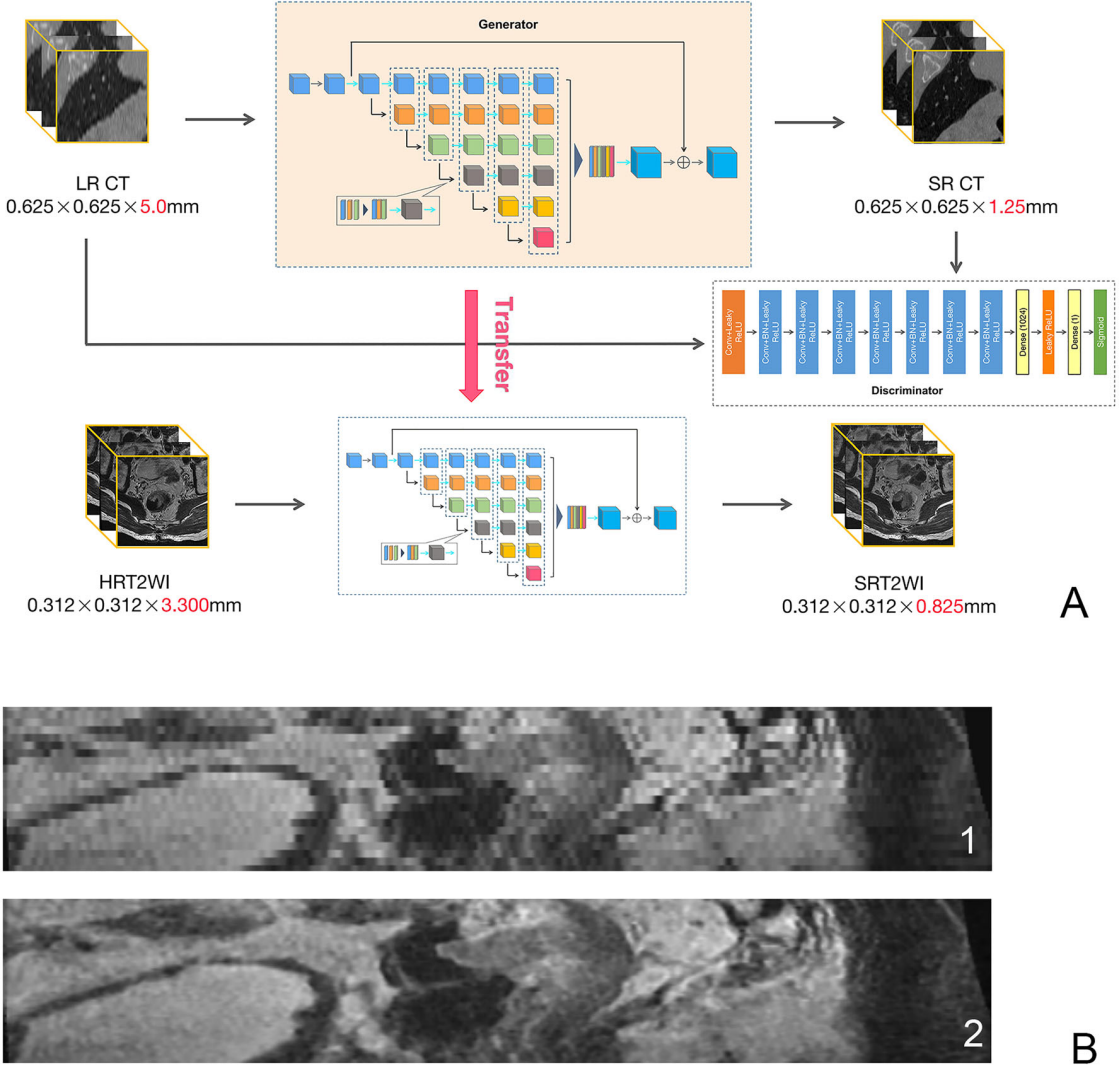
Diagram of transfer learning to obtain the fourfold enhanced super-*z* images (**A**). First, Gaussian noise was added to the CT images to reduce the out-plane resolution with a factor of 4 to generate a new low-resolution (LR) image. Then, the LR and synthetic high-resolution (HR) image pairs were used to train a lightweight parallel generative adversarial network (GAN) model. Finally, the trained model was applied to HRT2WI by transfer learning. As the *z*-resolution improved, the sagittal GAN-SRT2 images (B2) are visually near-exact to the original HRT2 images (B1), but less blurry, with finer texture and sharper edges

On the basis of HRT2WI, we used a deep-transfer-learning network (Fig. 2A) to enhance the *z*-resolution, improving the spacing from 0.312 × 0.312 × 3.300 mm to 0.312 × 0.312 × 0.825 mm. The newly developed images were defined as SRT2WI (Fig. 2B). The quality of the SRT2WI images was assessed by structure similarity (SSIM) and normalized root mean square error (NRMSE). The results were 0.974 and − 0.360%, respectively, manifesting very subtle structural or intensity changes, but effectively improved resolution. Details of the synthesis framework and evaluation formulations are provided in Supplementary materials 2.

Two expert abdominal radiologists (with 22- and 25-year experience) reviewed all the images in consensus while they were blinded to the pathological information. Disagreement on interpretations was resolved by discussion. The MRI T-staging was evaluated according to the following criteria based on the 8^th^ AJCC TNM classification [29]: on HRT2WI, T1/2 tumors were confined to the hyperintense submucosa but not beyond the hypointense muscularis propria, and tumors with mesorectal fat layer infiltration or direct invasion of surrounding structures indicated LARC.

### Image segmentation and feature extraction

The ITK-SNAP (version 3.8.0, www.itksnap.org) software was utilized for manual segmentation on each HRT2WI and SRT2WI slice. Intestinal contents or air was carefully avoided when drawing the contours. We also excluded the top and bottom slices to reduce bias caused by partial volume effects. Firstly, MRI images of 40 subjects (10 cases of stage T1, T2, T3, and T4 each) were randomly selected, and then two radiologists (with 9- and 15-year experience) independently outlined the entire tumor as they were blinded to all the clinical information except the tumor location. Radiologist 1 repeated the process 2 weeks later. The intra-/inter-observer variability of the radiomics features was assessed by inter-class correlation coefficient (ICC) test (Supplementary materials 3). An ICC above 0.80 was considered of good reproducibility. Radiologist 1 finished the delineation of the remaining subjects. The volume of interest (VOI) was reconstructed based on the ROIs for each patient.

**Fig. 3:**
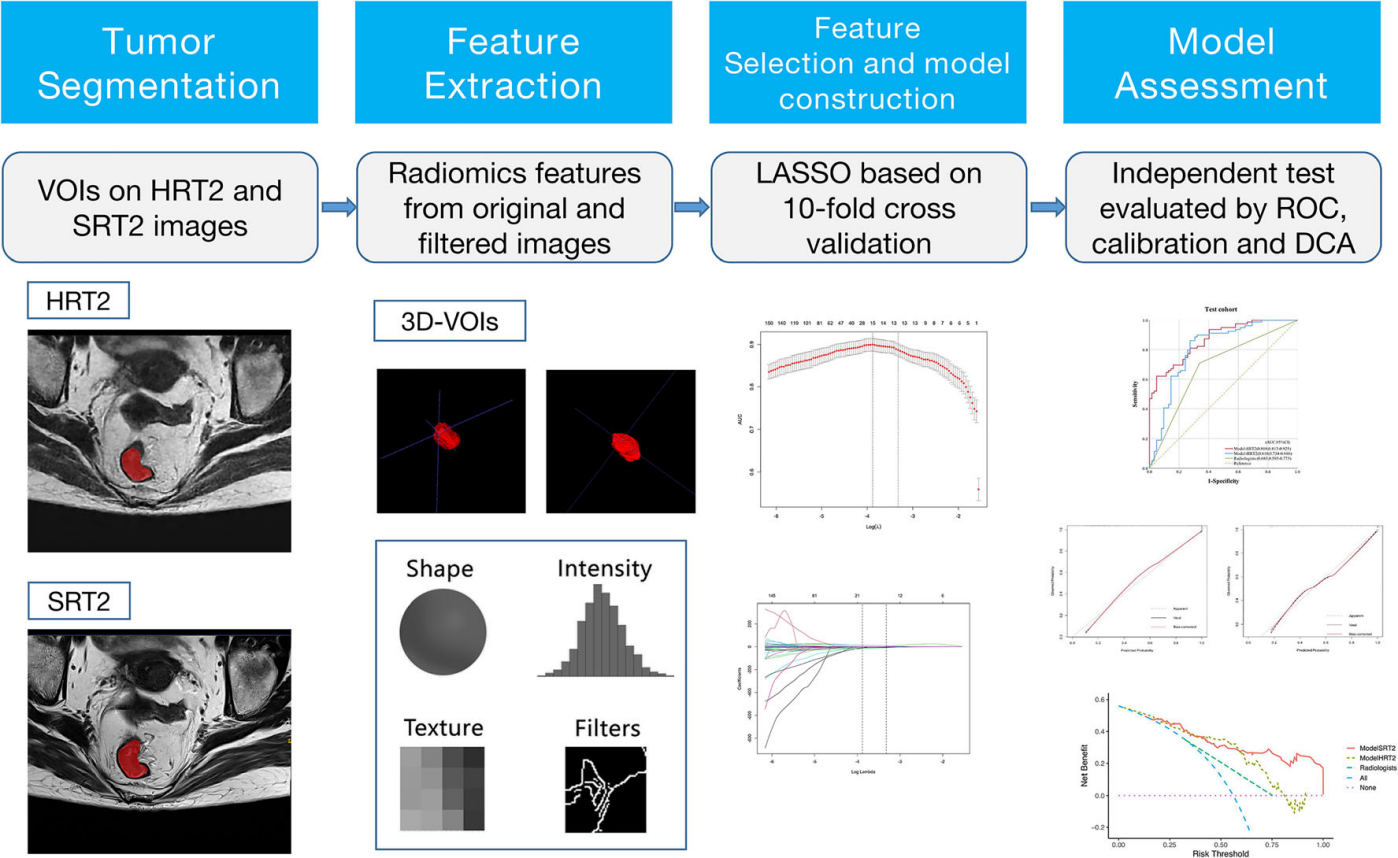
The radiomics flowchart of this study

Pre-processing procedures were undertaken to facilitate feature reproducibility and reduce the influence of different acquisition parameters. All MRI images and segmentations were resampled to a standardized voxel size of 1 × 1 × 1 mm^3^, and the voxel intensity values were discretized with a fixed bin width of 25 units. Afterwards, quantitative radiomics features were respectively extracted from segmented HRT2WI and SRT2WI via pyradiomics version 2.2.0 [30]. Feature extraction is illustrated in Fig. 3 as part of the whole radiomics workflow.

### Feature selection and radiomics model construction

Feature selection was performed on the training cohort through a two-step approach. First, the Kendall correlation coefficient was calculated to evaluate the relevance between each feature and the tumor T-category. Salient features with coefficient ≥ 0.8 were retained as candidate features [31]. Subsequently, the Least Absolute Shrinkage and Selection Operator (LASSO) regression algorithm was applied for further dimensionality reduction and optimal feature selection. In this procedure, the one-standard error of the minimum criteria (the 1-SE criteria) was used for tuning the regularization parameter (*λ*) and tenfold cross-validation-based feature selection. Finally, the optimal features with corresponding non-zero coefficients were linearly combined to obtain a radiomics score (Rad-score) for classification analysis.

### Performance assessment of the models

ROC curves were plotted, and the corresponding AUCs were calculated to assess the discrimination performance of the predictive models. Accuracy, sensitivity, and specificity were analyzed for each model at a cut-off value derived from the maximum Youden index. The DeLong test was conducted to compare the ROCs. Calibration curves and the Hosmer-Lemeshow test were used to evaluate the apparent performance; a non-significant test statistic indicated good calibration. Clinical utility of the models was measured by decision curve analysis (DCA) through quantifying the net benefits at different threshold probabilities in the testing cohort.

### Statistical analysis

Continuous variables were described as mean ± standard deviation and compared by a *t* test or Mann-Whitney *U* test. Categorical variables were summarized as frequencies and percentages using the chi-square test or Fisher’s exact test. A *p* value < 0.05 was considered statistically significant. Ninety-five percent confidence intervals (CIs) were estimated by 1000-replicate bootstrapping. Statistical analysis was performed with SPSS software (version 26.0, IBM) and R package (version 3.5.1, http://www.Rproject.org).

## Results

### Clinical characteristics and visual assessment by conventional HRT2WI

A total of 706 patients (mean age 65.39 ± 10.481 years) were included in this study. Male patients (*n* = 437, 61.9%) and moderately differentiated patients (*n* = 492, 69.7%) held the majority. Early-stage patients (*n* = 287, 40.7%) were around two-thirds of the T3/4 patients (*n* = 419, 59.3%), and they were distributed similarly in the training and testing cohorts (*p* = 0.370). There was no significant difference in all clinicopathological characteristics (all *p* > 0.05) between the training and testing cohorts, as summarized in Table 2. The predictive accuracy of the radiologists’ visual diagnosis based on routine HRT2WI was 0.701 in the training cohort and 0.685 in the testing cohort (*p* = 0.230).

**Table 2:** Clinicopathological characteristics of patients in the training and testing cohorts

	Total *n* percentile (%)	Training cohort (80%) *n* percentile (%)	Testing cohort (20%) *n* percentile (%)	*p*
Age (mean ± SD, years)	65.39 ± 10.481	65.14 ± 10.592	66.36 ± 10.000	0.217
Gender				0.267
Male	437 (61.9)	344 (60.9)	93 (66.0)	
Female	269 (38.1)	221 (39.1)	48 (34.0)	
BMI	23.083 ± 3.089	23.20 ± 3.034	22.998 ± 3.134	0.536
Tumor size (mean ± SD, cm)	3.76 ± 1.525	3.76 ± 1.515	3.76 ± 1.567	0.995
Tumor location				0.945
Low	143 (20.2)	113 (20.0)	30 (21.3)	
Middle	304 (43.1)	244 (43.2)	60 (42.5)	
High	259 (36.7)	208 (36.8)	51 (36.2)	
Pathological T stage				0.243
pT1	105 (14.9)	77 (13.6)	28 (19.9)	
pT2	182 (25.8)	148 (26.2)	34 (24.1)	
pT3	323 (45.8)	265 (46.9)	58 (41.1)	
pT4	96 (13.5)	75 (13.3)	21 (14.9)	
Differentiation grade				0.719
Well	105 (14.7)	85 (15.0)	25 (17.7)	
Moderate	492 (69.7)	397 (70.3)	95 (67.4)	
Poor	109 (15.6)	83 (14.7)	21 (14.9)	
Pathological LVI status				0.144
Negative	438 (62.0)	343 (60.7)	95 (67.4)	
Positive	268 (38.0)	222 (39.3)	46 (32.6)	
Pathological PNI status				0.169
Negative	628 (89.0)	498 (88.1)	130 (92.2)	
Positive	78 (11.0)	67 (11.9)	11 (7.8)	
Pathological EMVI status				0.858
Negative	633 (89.7)	506 (89.6)	127 (90.1)	
Positive	73 (10.3)	59 (10.4)	14 (9.9)	
Tumor deposit				0.807
Negative	520 (73.7)	415 (73.5)	105 (74.5)	
Positive	186 (26.3)	150 (26.5)	36 (25.5)	
Gross histopathology				0.593
Expanding	285 (40.4)	227 (40.2)	58 (41.1)	
Infiltrating	66 (9.3)	50 (8.8)	16 (11.3)	
Ulcering	355 (50.3)	288 (51.0)	67 (47.5)	
CEA level				0.545
Normal (< 5 ng/ml)	496 (70.3)	394 (69.7)	102 (72.3)	
Elevated (≥ 5 ng/ml)	210 (29.7)	171 (30.3)	39 (27.7)	
CA 19-9 level				0.996
Normal (< 37 U/ml)	651 (92.2)	521 (92.2)	130 (92.2)	
Elevated (≥ 37 U/ml)	55 (7.8)	44 (7.8)	11 (7.8)	

*SD* standard deviation, *LVI* lymphovascular invasion, *PNI* perineural invasion, *EMVI* extramural venous invasion, *CEA* carcinoembryonic antigen, *CA19-9* carbohydrate antigen 19-9

### Radiomics feature processing and model development

Altogether, 1688 radiomics features were extracted from each T2WI sequence. The obtained features, including 18 first-order features, 14 shape features, and 75 high-order texture features after applying 18 image filters for each, resulted in 1688 (i.e., 18 × [75 + 18] + 14) feature-filter combinations. The mean inter- and intra-observer reliabilities were 0.811 (95% CI 0.726–0.896) and 0.839 (95% CI 0.776–0.902), indicating excellent consistency in radiomics features. Among the 1688 features, a total of 143 and 152 features respectively from HRT2WI and SRT2WI were retained according to their Kendall correlation coefficients. The optimal *λ* (*λ*_H_ = 0.01541968, *λ*_S_ = 0.02163163) with transformed log(*λ*)s [log(*λ*_H_) = − 4.1721, log(*λ*_S_) = − 3.8336] was respectively selected. Eventually, 13 features in HRT2WI and 8 in SRT2WI were chosen to build the radiomics models, which were defined as model_HRT2_ and model_SRT2_, respectively. Corresponding Rad-score equations and further details on feature extraction are available in Supplementary materials 4.

**Fig. 4:**
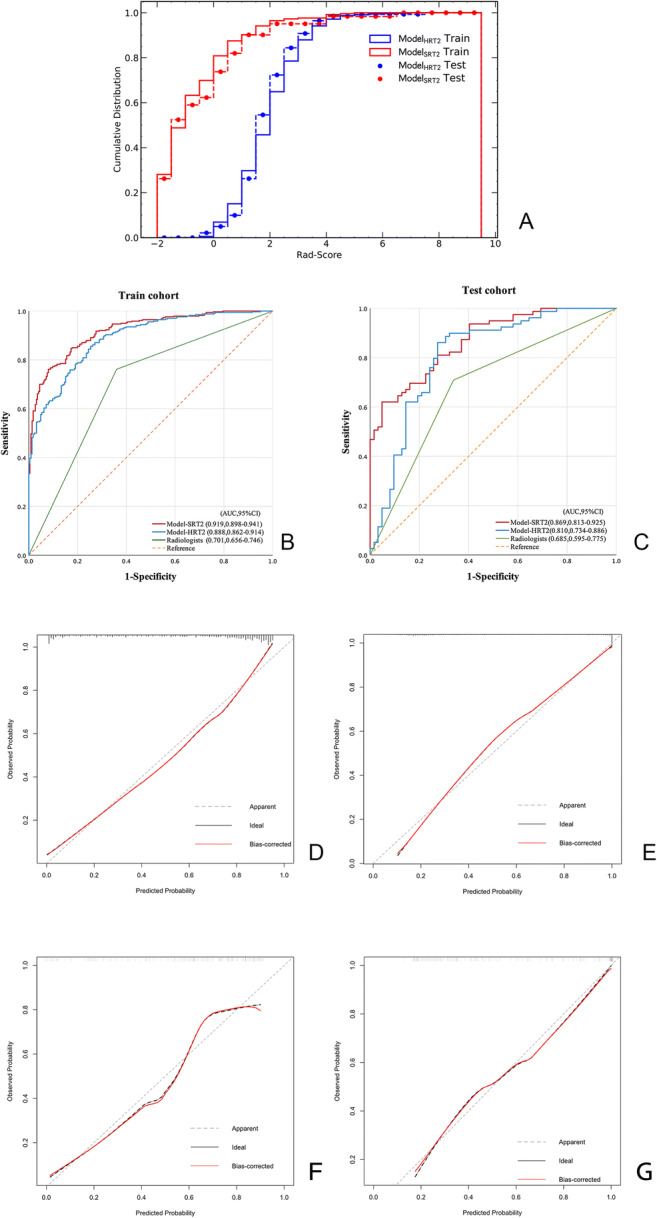
Evaluation of the models. **A** Cumulative distribution curves of the Rad scores. The ROCs for the training (**B**) and the test (**C**) cohorts. The calibration curves demonstrated good calibration of the models in both cohorts (**D**–**G**)

The Rad scores were significantly higher in T3/4 patients than in T1/2 patients in both model_HRT2_ (training: 2.754 ± 1.100 vs. 1.289 ± 0.721, testing: 2.506 ± 0.997 vs. 1.531 ± 0.915, both *p* < 0.001) and model_SRT2_ (training: − 3.055 ± 0.806 vs. − 1.022 ± 1.746, testing: − 2.945 ± 0.901 vs. − 0.930 ± 1.878, both *p* < 0.001). Furthermore, the cumulative distribution of Rad scores was similar between training and testing cohorts in both models (Fig. 4A), indicating that no overfitting was observed.

### Predictive performance of the models

The AUCs of model_HRT2_ and model_SRT2_ were 0.888 (95% CI 0.862–0.914) and 0.919 (95% CI 0.898–﻿0.941), respectively, in the training cohort (Fig. 4B). In the testing cohort, model_SRT2_ yielded an AUC of 0.869 (95% CI 0.813–0.925), suggesting a better performance compared to model_HRT2_ (0.810, 95% CI 0.734–0.886). Both radiomics models exhibited more favorable performance in differentiating T1/2 and T3/4 than the expert radiologists (AUC = 0.685, 95% CI 0.595–0.775) (Fig. 4C). Further evaluation regarding accuracy, sensitivity, and specificity is shown in Table 3. The DeLong test showed significant difference among the ROCs of model_SRT2_, model_HRT2_, and radiologists (all *p* < 0.05).

**Table 3:** Predictive performance of model_HRT2_ and model_SRT2_

		AUC	Sensitivity	Specificity	Accuracy
Testing cohort	Model_HRT2_	0.810 (0.734–0.886)	0.895 (0.831–0.959)	0.701 (0.604–0.798)	0.773 (0.672–0.874)
	Model_SRT2_	0.869 (0.813–0.925)	0.711 (0.627–0.795)	0.931 (0.917–0.945)	0.833 (0.779–0.887)

*AUC* area under the curve

For model_HRT2_ and model_SRT2_, the calibration curves (Fig. 4D–G) and the Hosmer-Lemeshow test results (model_HRT2_: *p*_training_ = 0.501, *p*_testing_ = 0.316; model_SRT2_: *p*_training_ = 0.955, *p*_testing_ = 0.397) showed no departures from perfect fit in both cohorts.

### Clinical utility

**Fig. 5:**
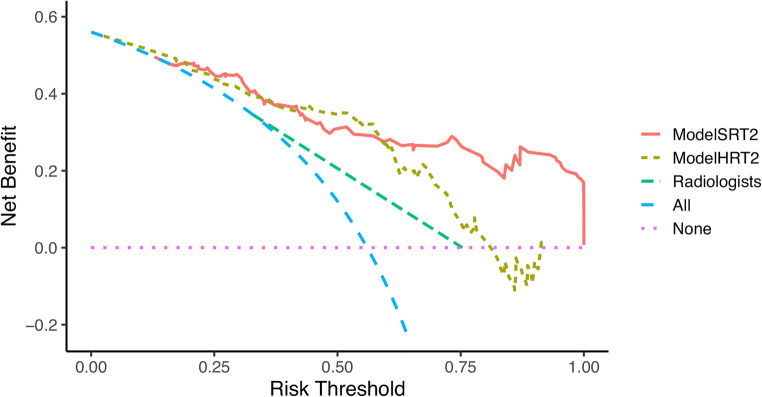
Decision curve analysis for the predictive models. The *y*-axis stands for the net benefit, and the *x*-axis represents the threshold probability. The decision curves indicated that the application of model_SRT2_ and model_HRT2_ for preoperative T-staging assessment gains more net benefit than the all/none-intervention strategy at a probability threshold of 0.18–1.00 and 0.03–0.81, respectively, while the diagnostic model of radiologists works at a probability threshold of 0.35–0.75. The three models all showed net benefit for T-staging, and model_SRT2_ is the most preferred to be adopted for RC patients

The DCA (Fig. 5) indicated that model_SRT2_ gained more net benefit than model_HRT2_, and the application of both radiomics models improved the clinical benefit for preoperative T-staging assessment in RC compared to expert radiologists.

## Discussion

This study developed and independently validated tumor-derived radiomics models based on HRT2WI and SRT2WI for preoperative T-staging prediction in RC. Compared to expert radiologists and model_HRT2_, model_SRT2_ exhibited superior predictive performance with enhanced image quality, indicating the potential to assist clinicians in selecting the optimal initial treatment strategy for RC patients.

Accurate assessment of T-staging is crucial for the decision of whether to conduct nCRT before surgery [8]. In addition, the primary T-staging plays a significant role in post-treatment evaluation as baseline reference [9, 32]. Currently, MRI is recommended as standard for local T-staging of RC, while the accuracy is ungratified [11–14]. Hence, an alternative approach is needed to facilitate the pretreatment assessment. Owing to high-throughput data digging, radiomics provides extensive additional information for clinical use [17]. A few studies have tried to adopt radiomics analysis in RC T-staging. Sun et al [33] conducted a pilot study with a predictive AUC of 0.852 in the training dataset. Though this study found that preoperative radiomics features were feasible to identify local T-staging, it lacked an independent test. In the most recent study that retrospectively enrolled 268 patients, Lin et al [34] selected 9 optimal radiomics features and achieved an AUC of 0.807. We extracted massive radiomics features from VOIs of the original and filtered images; thus, more comprehensive information on intra-tumor heterogeneity might be characterized. In our study, the diagnostic performance of model_HRT2_ was similar to or even slightly higher than those of the previously reported models. The above studies indicate that HRT2WI-based radiomics models exhibited equally good performance.

Nevertheless, considering the physical process of HRT2WI, the in-plane resolution is higher than that out-of-plane [35]. As a result, spatial resolution along the *z*-direction (*z*-resolution) is anisotropic, which may have an adverse effect on 3D-based radiomics features. This issue could be ameliorated by SR methods [26, 35, 36]. Among various SR methods proposed for medical imaging, DL-SR was favored for its ability to overcome limitations of high-frequency information loss and edge blurring as well as increase resolution [37, 38]. In recent years, few researches have laid the foundation for employing DL-SR in radiomics analysis. Fan et al [39] explored the usefulness of the 2D SR neural network for resolution improvement and image-based diagnosis. Farias et al [24] found that generative adversarial network (GAN) SR increased the robustness of the most important radiomics features. These two proof-of-concept studies have revealed encouraging potential to further apply DL-SR to the practice of radiomics analysis, while validation for clinical utility by workflow is still warranted. In contrast to the preliminary studies mentioned above, we developed the 3D GAN-based model_SRT2_ and focused on its clinical application for RC T-staging prediction. Our model_SRT2_ achieved superior diagnostic performance (AUC 0.869) compared with previous radiomics models. One explanation could be that the DL-SR method performs high-precision interpolation by combining contextual information, and consequently, the SR images may provide more encrypted information for radiomics analysis which is deemed to be quite sensitive in recognizing subtle changes in tumor morphology and pathophysiology. Furthermore, the process of feature extraction and stability of the features in model_SRT2_ are more resilient to imaging anisotropy along the *z*-direction compared to that in model_HRT2_.

In our study, both radiomics models outperformed the expert radiologists in terms of accuracy. We noticed that radiologists’ misjudgment was mainly caused by confusing T2 and T3 tumors. This might be explained by the difficulty of visually identifying micro-infiltration and classifying indistinguishable radiological characteristics [40]. Additionally, radiologists might be more prone to suspect a tumor of LARC due to the fear of negative consequences of missing mesorectal invasion, especially for borderline tumors. Intriguingly, a higher resolution can provide better visibility of the rectal wall, whereas the research results on whether this could help radiologists improve the diagnostic accuracy of discriminating T2 from T3 tumors are controversial and contradictory. Maas et al [16] proposed the paradox that more overstaging cases and lower inter-observer agreement arose as resolution improved, while Kim et al [15] reported that the resolution did not affect the staging results for radiologists. Even so, both authors agreed that either mis-staging or the low-moderate inter-observer agreement was hard to manage owing to subjective influence even among senior radiologists [15, 16]. In this regard, radiomics models have shown better and much more stable performance by virtue of the objectivity advantage of artificial intelligence. This explains why model_SRT2_ could make good use of SR and produce a more objective and balanced outcome compared to subjective assessment by radiologists. In addition, it is noteworthy that our T-staging cohort is the largest to date, and therefore, our models are more reliable.

Several limitations exist in this study. First, the radiomics models were constructed based on manual delineation. While model_SRT2_ did achieve better performance, the application of SR technology increases the workload of segmentation. In the future, the combination of automatic segmentation and radiomics analysis will provide more efficiency and robustness for SR models. Second, the handcrafted radiomics features are subject to individual experience and perception of the radiologists. In subsequent research, DL-based automatic feature selection may capture more efficient features and minimize the subjective interference [41]. Third, it was a retrospective single-center study. A prospective design with external validation is warranted for further investigation.

In conclusion, the study presented a DL-based 3D SR radiomics model, which outperformed the conventional HRT2 radiomics model and expert radiologists in predicting tumor T-staging and identifying nCRT candidates among RC patients. So far, this is the first time the DL-based 3D SR method has been applied to a radiomics analysis for clinical decision support. We believe that the clinical utility of radiomics by means of SR imaging would be maximized through further validation.

## Supplementary information

ESM 1 (DOCX 183 kb)

## Abbreviations

3D: Three dimensional
AJCC: American Joint Commission on Cancer
CA19-9: Carbohydrate antigen 19-9
CEA: Carcinoembryonic antigen
CI: Confidence interval
CNN: Convolutional neural network
DCA: Decision curve analysis
DL: Deep learning
EMVI: Extramural venous invasion
GAN: Generative adversarial network
HR: High resolution
ICC: Intra (inter)-observer correlation coefficient
LARC: Locally advanced rectal cancer
LASSO: The Least Absolute Shrinkage and Selection Operator
LVI: Lymphovascular invasion
nCRT: Neoadjuvant chemoradiotherapy
NRMSE: Normalized root mean square error
PNI: Perineural invasion
RC: Rectal cancer
SR: Super-resolution
SSIM: Structure similarity
T1WI: T1-weighted imaging
T2WI: T2-weighted imaging
VOI: Volume of interest

## Declarations

### Guarantor

The scientific guarantor of this publication is Professor Jihong Sun.

### Statistics and biometry

No complex statistical methods were necessary for this paper.

### Methodology

• retrospective

• diagnostic or prognostic study

• performed at one institution
