# Supplementary material for: Deep-learning-based 3D super-resolution MRI radiomics model: superior predictive performance in preoperative T-staging of rectal cancer
Source: Eur Radiol. 2022 Jun 21;33(1):1–10. doi: 10.1007/s00330-022-08952-8 (PMC9755091; doi:10.1007/s00330-022-08952-8)
Supplement: Supplementary file 1 — (DOCX 183 kb) [file 330_2022_8952_MOESM1_ESM.docx]

***Supplementary Materials 1***

**Definitions of histopathological T-staging**

Postoperative histopathological T-staging was determined according to the American Joint Commission on Cancer (AJCC, 8^th^ Edition) classification^[1]^ as follows:

T1: Tumor invades the submucosa (through the muscularis mucosa but not into the muscularis mucosae);

T2: Tumor invades the muscularis propria;

T3: Tumor invades through the muscularis propria into pericolorectal tissues;

T4: Tumor invades the visceral peritoneum or adheres to adjacent organ structure;

***Supplementary Materials 2***

**The structure details of z-SR framework**

The z-SR framework consists of two modules, generator and discriminator. A VolumeNet^[2]^-derived parallel-connected architecture was employed for the generator, which served the function of feature extraction, global residual connectivity, parallel connectivity module and feature aggregation. In the generator, thirty-two sets of 3×3×3 convolutional kernels were used to extract shallow features at the head, the parallel connected modules with N convolutional layers and feature aggregation blocks formed the main part, and a global residual connection structure was applied in the tail to effectively avoid the gradient disappearance problem. The discriminator network, which is the same as SRGAN^[3]^, mainly included a convolutional kernel of size 3×3×3, followed by batch-normalization (BN) layers and Leaky rectified linear unit (ReLU, α=0.2) as the activation function, and a self-attentive layer in the fourth layer^[4]^. Finally, the probability of true or false is output by a fully connected layer and a sigmoid function.

**Model implementation details**

For each input (low-resolution image) X in the training cohort, the following objective function was optimized:


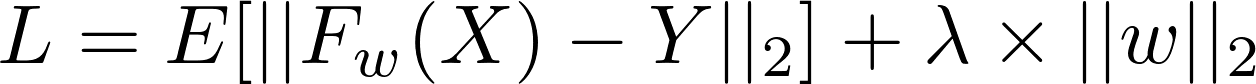


Where the first term is Mean square error (MSE) loss, and the second term is regularization loss. Y is the output corresponding to X, *w* represents the model parameters, and λ is a weighting factor that balances the MSE loss and the regularization loss.

During the training phase, the Adam optimization method was used with batch size 16, and λ was set to 0.001. The learning rate was modified using the exponential decay approach, with the starting value set to be 0.0001. All the steps were implemented using Python 3.7 and PyTorch 1.7 on a Ubuntu workstation with one NVIDIA GeForce RTX 1080 GPU.

**Evaluation of DL-based 3D SR images**

We used the structure similarity (SSIM)^[5]^ and normalized root mean square error (NRMSE) to measure the quality of the synthesized SR images. SSIM and NRMSER are defined as follows:


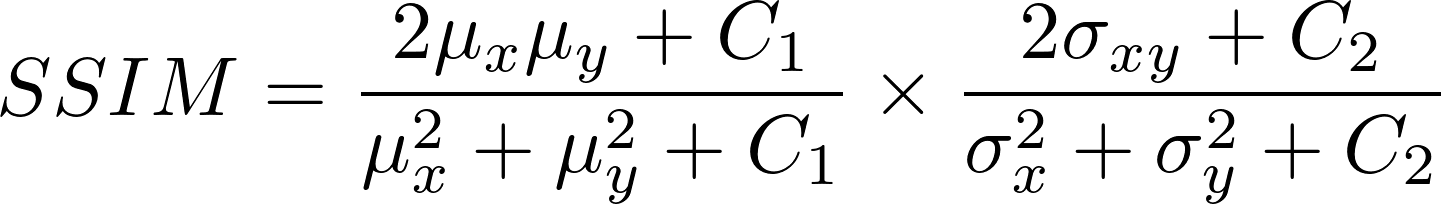


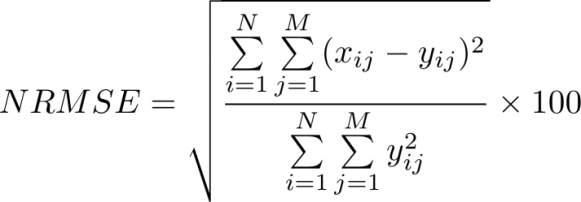

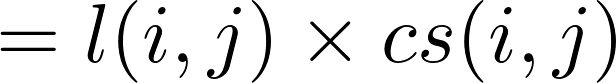


Where C_1_ and C_2_ are constants, µ_x_, µ_y_, σ_x_, σ_y_, and σ_xy_ are the image statistics calculated in the patch centered at pixel (*i,j*), *x* and *y* represent HRT2WI and SRT2WI, respectively. The SSIM value ranges from 0 to 1. The closer the two images are to each other, the closer the SSIM value is to 1.

***Supplementary Materials 3***

**The formulation of ICC**

The formulation of ICC we used was


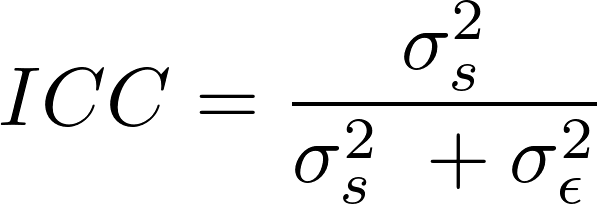


Where δ_s_ indicates variance caused by differences between the delineations; and δ_ε_ indicates variance caused by differences between the points in delineations^[6]^.

***Supplementary Materials 4***

**Radiomics Features**

1. List of Feature category numbers and abbreviations

| Shape (n=14) | This group of features included descriptors of the three-dimensional size and shape of the ROI. |
| --- | --- |
| Firstorder (n=18) | First-order statistics described the distribution of voxel intensities within the image region defined by the mask through commonly used and basic metrics. |
| GLCM (n=24) | A Gray Level Co-occurrence Matrix (GLCM) of size described the second- order joint probability function of an image region constrained by the mask |
| GLSZM (n=16) | A Gray Level Size Zone (GLSZM) quantified gray level zones in an image. A gray level zone is defined as the number of connected voxels that share the same gray level intensity. |
| GLRLM (n=16) | A Gray Level Run Length Matrix (GLRLM) quantified gray level runs, which are defined as the length in number of pixels, of consecutive pixels that have the same gray level value. |
| NGTDM (n=5) | A Neighboring Gray Tone Difference Matrix (NGTDM) quantifies the difference between a gray value and the average gray value of its neighbors. |
| GLDM (n=14) | A Gray Level Dependence Matrix (GLDM) quantified gray level dependencies in an image. |

Source: https://pyradiomics.readthedocs.io/en/2.2.0/features.html

1. List of all 18 image filters applied prior to feature extraction, including the identity filter (original).

| Image filters |
| --- |
| exponential |
| lbp-3D-k |
| lbp-3D-m1 |
| lbp-3D-m2 |
| log-sigma-2–0-mm-3D |
| log-sigma-3–0-mm-3D |
| logarithm |
| Original |
| Square |
| squareroot |
| wavelet-HHH |
| wavelet-HHL |
| wavelet-HLH |
| wavelet-HLL |
| wavelet-LHH |
| wavelet-LHL |
| wavelet-LLH |
| wavelet-LLL |

Source: https://pyradiomics.readthedocs.io/en/2.2.0/features.html

1. According to the Kendall correlation coefficients, 143 and 152 features were screened for subsequent feature selection from each 1688 features. They were:

143 features (43 GLCM features, 29 GLSZM features, 17 GLRLM features, 10 NGTDM features, 5 GLDM features, and 39 first-order features) from HRT2WI; and 152 features ( 45 GLCM features, 31 GLSZM features, 22 GLRLM features, 9 NGTDM features, 4 GLDM features, and 41 first-order features) from SRT2WI.

4. The Radscore of each selected feature was calculated and converted to a probability (range 0–1) of T-staging category prediction for each subject by using the following sigmoid function:


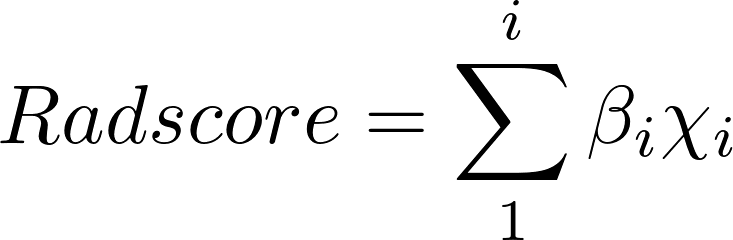


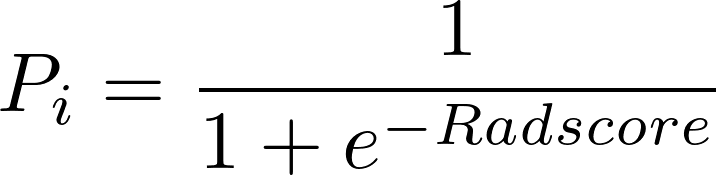


X_i_ refers to the input radiomics features, β_i_ indicates the coefficients, and P represents the probability of corresponding T-staging. e is Euler number (e=2.71828).

Model_HRT2_ and Model_SRT2_ were respectively established with Rad-scores calculated as follows:

Rad-score_HRT2WI_＝0.5520521322*lbp-3D-k_firstorder_RobustMeanAbsoluteDeviation＋0.4259271205*log-sigma-2-0-mm-3D_glcm_Correlation＋0.0009062525*log-sigma-3-0-mm-3D_firstorder_10Percentile＋-0.3465683346*log-sigma-3-0-mm-3D_glcm_DifferenceEntropy＋1.0841444997*log-sigma-3-0-mm-3D_glcm_Idm＋0.0211943094*wavelet-HLL_firstorder_Kurtosis＋-2.0278828837*wavelet-LHH_glcm_Imc2＋0.0006037942*lbp-3D-m1_glrlm_RunLengthNonUniformity＋0.0008197538*log-sigma-2-0-mm-3D_firstorder_Median＋-0.7530076687*log-sigma-3-0-mm-3D_firstorder_Skewness＋0.0372524168*wavelet-HLL_firstorder_Median＋0.0090296127*wavelet-LHL_firstorder_Maximum＋0.0001047404*wavelet-LHL_glszm_SizeZoneNonUniformity

Rad-score_SRT2WI_＝12.923271579*wavelet-LHL_glcm_Idn＋-8.992622429*lbp-3D-k_glszm_SmallAreaEmphasis＋0.002009458*lbp-3D-m1_glrlm_RunLengthNonUniformity＋-0.774987453*lbp-3D-m2_firstorder_90Percentile＋-1.128136625*log-sigma-2-0-mm-3D_glcm_Imc1＋-18.112073461*wavelet-HLL_glcm_Imc1＋-1.33499684*wavelet-LHH_firstorder_Skewness＋0.00164919*wavelet-LHH_glszm_GrayLevelNonUniformity

***Supplementary References***

1 Amin MB, Greene FL, Edge SB et al (2017) The Eighth Edition AJCC Cancer Staging Manual: Continuing to build a bridge from a population-based to a more "personalized" approach to cancer staging. CA Cancer J Clin 67:93-99

2 Li Y, Iwamoto Y, Lin L, Xu R, Tong R, Chen YW (2021) VolumeNet: A Lightweight Parallel Network for Super-Resolution of MR and CT Volumetric Data. IEEE Trans Image Process 30:4840-4854

3 Ledig C, Theis L, Huszar F et al (2017) Photo-Realistic Single Image Super-Resolution Using a Generative Adversarial Network2017 IEEE Conference on Computer Vision and Pattern Recognition (CVPR), pp 105-114

4 Zhang H, Goodfellow I, Metaxas D, Odena A (2019) Self-Attention Generative Adversarial Networks. In: Kamalika C, Ruslan S, (eds) Proceedings of the 36th International Conference on Machine Learning. PMLR, Proceedings of Machine Learning Research, pp 7354--7363

5 Wang Z, Bovik AC, Sheikh HR, Simoncelli EP (2004) Image quality assessment: from error visibility to structural similarity. IEEE Trans Image Process 13:600-612

6 Taha AA, Hanbury A (2015) Metrics for evaluating 3D medical image segmentation: analysis, selection, and tool. BMC Med Imaging 15:29
